# Effect of Depth of Cut and Number of Layers on the Surface Roughness and Surface Homogeneity After Milling of Al/CFRP Stacks

**DOI:** 10.3390/ma18010206

**Published:** 2025-01-06

**Authors:** Elżbieta Doluk, Anna Rudawska, Stanisław Legutko

**Affiliations:** 1 Department of Production Computerisation and Robotisation, Faculty of Mechanical Engineering, Lublin University of Technology, Nadbystrzycka 36, 20-618 Lublin, Poland; a.rudawska@pollub.pl; 2 Faculty of Mechanical Engineering, Poznan University of Technology, Pl. Marii Skłodowskiej-Curie 5, 60-965 Poznań, Poland; stanislaw.legutko@put.poznan.pl

**Keywords:** Al/CFRP sandwich structures, machining, milling, surface roughness, surface homogeneity index

## Abstract

A multilayer structure is a type of construction consisting of outer layers and a core, which is mainly characterized by high strength and specific stiffness, as well as the ability to dampen vibration and sound. This structure combines the high strength of traditional materials (mainly metals) and composites. Currently, sandwich structures in any configurations (types of core) are one of the main directions of technology development and research. This paper evaluates the surface quality of II- and III-layer sandwich structures that are a combination of aluminum alloy and CFRP (Carbon Fiber-Reinforced Polymer) after the machining. The effect of depth of cut (a_e_) on the surface roughness of the II- and III-layer sandwich structures after the milling process was investigated. The surface homogeneity was also investigated. It was expressed by the I_Ra_ and I_Rz_ surface homogeneity indices formed from the Ra and Rz surface roughness parameters measured separately for each layer of the materials forming the sandwich structure. It was noted that the lowest surface roughness (Ra = 0.03 µm and Rz = 0.20 µm) was obtained after the milling of the II-layer sandwich structure using a_e_ = 0.5 mm, while the highest was obtained for the III-layer structure and a_e_ = 1.0 mm (Ra = 1.73 µm) and a_e_ = 0.5 mm (Rz = 10.98 µm). The most homogeneous surfaces were observed after machining of the II-layer structure and using the depth of cut a_e_ = 2.0 mm (I_Ra_ = 0.28 and I_Rz_ = 0.06), while the least homogeneous surfaces were obtained after milling of the III-layer structure and the depths of cut a_e_ = 0.5 mm (I_Ra_ = 0.64) and a_e_ = 2.0 mm (I_Rz_ = 0.78). The obtained results may be relevant to surface engineering and combining hybrid sandwich structures with other materials.

## 1. Introduction

Structural composites are now one of the more commonly used material groups. These are constructions formed by combining outer layers (faces) and an inner layer (core) made of different materials. Such a combination of materials makes it possible to obtain a structure with high strength and stiffness (high stiffness- and strength-to-weight ratio) which is used in the marine, aerospace, armament, and automotive industries, among others [1,2,3]. One obstacle to the use of sandwich structures is their machining—because of their heterogeneous nature, these materials are considered difficult to machine. This is due to the combination of materials sometimes with extremely different properties, such as metal alloys and FRP (Fiber-Reinforced Polymer), which are characterized by high anisotropy [4]. Many works focus on the machinability of FRP composites [5,6,7] and the quality of assembly holes after machining of sandwich structures with different faces materials and different core types [8,9]. More and more research is being conducted on the feasibility of a helical milling process [10,11] or other unconventional technologies for machining of sandwich materials [12,13]. There is also a lot of work on the machinability of honeycomb core sandwich structures [14,15,16]. Today, hybrid sandwich structures (polymer composite/metal alloy) are becoming increasingly important. The most common combinations are structures built with FRP composites and metal alloys in various configurations, such as combinations of CFRP (Carbon Fiber-Reinforced Plastics) and aluminum alloy (Al/CFRP or CFRP/Al), combinations of titanium alloys and CFRP (CFRP/Ti or Ti/CFRP), or a combination of all these materials (Al/CFRP/Ti) [17]. In many industries, strict tolerance ranges are placed on the quality of machined parts. The determination of surface roughness, especially for metallic materials, is needed to determine the impact of surface microstructure on the durability and reliability of structures [18,19]. It is assumed that for machine parts and equipment used in industry, the Ra surface roughness parameter of 3.2 µm or less is required [20,21]. Assessing surface quality by 2D surface roughness parameters (e.g., Ra, Rz) for sandwich structures may not be meaningful, as it does not take into account the complexity of defects arising in the polymer layer [22]. The most common operation that structural composites undergo is drilling (about 50%) [23]. In addition, these materials are also milled and cut using waterjet technology. Sandwich structures subjected to machining are more susceptible to damage than metallic materials. Fiber pullout, delamination, matrix cracking and smearing are typical defects that arise after machining of hybrid sandwich structures [24]. Voss et al. [25] studied the effects of cutting parameters (v_c_, f), tool geometry (clearance angle and rake angle), and fiber orientation (0°, 30°, 60°, 90°, 120°, 150°) on the surface quality of the CFRP after cutting. They showed that a high cutting speed (v_c_ = 200 m/min) and a moderate value of feed rate (v_f_ = 100 mm/min) allowed them to achieve the best surface quality and the lowest cutting forces. They also observed a positive effect of increasing the rake angle and clearance angle on the surface quality of the machined material. Jinyang et al. [26] studied the effect of the fiber orientation (0°, 45°, 90°, 135°) on cutting forces and cutting tool wear after orthogonal cutting of the CFRP/Ti stacks. They noted that higher cutting forces occur when machining of the metal layer. They confirmed that the orientation of the fibers in the polymer composite has a great influence on the surface quality of the machined material—lower angles contribute to reducing the occurrence of the surface damage. In [27], the effect of machining parameters and orientation of the composite reinforcement on the surface quality and the cutting temperature after the milling of the unidirectional CFRP was presented. It has been shown that the use of 0° and 90° and v_c_ = 200 m/min and v_c_ = 250 m/min made it possible to obtain the best surface integrity. El-Hofy et al. [28] examined the effect of machining conditions (v_c_ and f_z_), type of tool material (PCD and DLC), and cooling system (dry and chilled air) on surface roughness and surface integrity after slotting of the CFRP. Lu et al. [29] studied the effects of helix angle (20°, 40°, 60°) and the number of cutter blades (z = 2, 4, and 6), as well as cutting speed, feed speed, and radial depth of cut on surface topography and cutting forces after milling of the CFRP. They concluded that a higher value of a helix angle has a positive effect on the quality of the machined surface. The paper [30] presents the effects of the drilling in the CFRP/Ti6Al4V stacks using a minimal lubricating condition (MQL). The effects of the cooling method on the surface roughness and the hole diameter variation were studied. It was shown that the amount of machining fluid used affects tool life and hole quality. Nguyen-Dinh et al. [31] investigated the effect of cutting conditions (technological parameters, tool geometry, and cutting edge wear rate) on the formation of harmful particles dispersed during trimming of the CFRP. The results obtained when machining with a classical PCD tool were compared with an innovative tool with a specially adapted geometry. Based on the experiment, they noted that a lower cutting speed and a higher feed rate could reduce the dispersion of the harmful particles. In the study [32], the effects of the milling of the aluminum honeycomb structures are presented. The influence of cutting parameters on the values of the components of cutting force, chip morphology, and mesh size were determined. It has been proven that the cutting force and the depth of cut affect the chip size and that a properly designed 3D model of the process can correctly represent the milling process of this type of structure. The paper [33] examined the effect of machining conditions and tool geometry when milling of the Nomex honeycomb structure. It was noted that low feed rates increase cutting tool life and a limited number of teeth reduce cutting forces and machined surface quality. Li et al. [34] studied the surface roughness, kerf characteristics, and surface integrity after abrasive waterjet cutting of the hybrid sandwich structures. They showed that the machining strategy (CFRP/Al and Al/CFRP) affected the surface quality after the cutting process. They also noted that a power-to-speed ratio (Ė/u) could be used to determine the relationship between machining parameters, kerf width, and surface roughness. Although hybrid sandwich structures are often used in many industries (e.g., in aerospace as structural components), the cutting conditions for this type of material have not yet been sufficiently defined. Improperly selected cutting conditions (including technological parameters and type of cutting tool) result in reduced product quality and can lead to the exposure of the reinforcing fibers of the composite material. This increases the vulnerability of the structure to the external environment. Sandwich structures used in aviation are often combined with other materials to form large-scale constructions. Thus, a sufficiently low surface roughness is important to achieve a proper fit of the components. In addition, the aerospace industry often requires coatings (e.g., protective or decorative) on sandwich structures. Excessive surface roughness and surface inhomogeneity can also affect the operation of the structure and its visual aspects. This can also result in insufficient adhesion of the coating, thereby reducing its durability. Furthermore, in the case of sandwich structures bonded to other materials by adhesive bonding, post-machining surface inhomogeneity can affect the strength of the adhesive bond. Currently, there are no standards or regulations governing the acceptable surface quality of sandwich structures after machining. In addition, recommendations for cutting of hybrid sandwich structures are usually based on the results obtained for the machining of FRP. Thus, it seems reasonable to conduct a study to determine the effect of the depth of cut and the number of layers on the machined surface quality of the Al/CFRP stacks. Several papers have been published on the surface quality of the Al/CFRP stacks (Aluminum alloy/Carbon Fiber-Reinforced Plastics) after milling, but each time using variable machining conditions. In [35,36], the effects of cutting parameters (v_c_, f_z_, a_e_), tool coating (TiAlN-coated tool and non-coated tool) and milling strategy (Al/CFRP and CFRP/Al) during machining on material height deviation and cutting force after milling were presented. In [37], the effect of helix angle on the surface quality after cutting of an analogous sandwich structure was presented. In [38], the effect of feed rate (f_z_) and machining strategy during milling on surface roughness using a diamond cutter with straight teeth is presented. In [39], based on the obtained surface roughness parameters, a surface homogeneity index was proposed for this type of material. The present work is a continuation of ongoing research on machinability and possibilities for improving the machined surface quality of the Al/CFFRP stacks.

## 2. Materials and Methods

**Figure 1:**
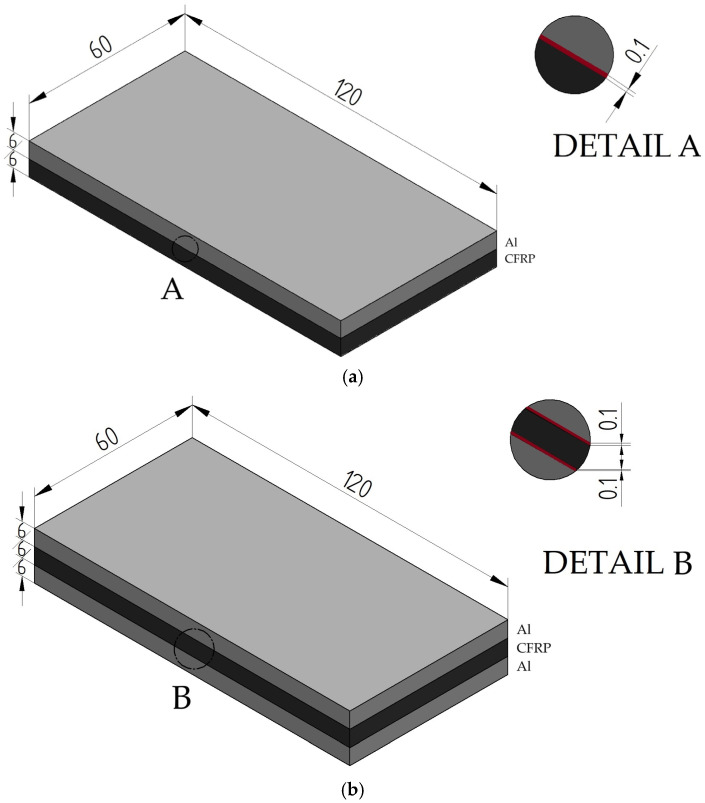
Shape and dimensions of the research object: (**a**) II-layer sandwich structure; (**b**) III-layer sandwich structure.

In this paper, surface quality of II- and III-sandwich structures after the machining process was studied. The structures were formed by combining two materials: 2024-T3 aircraft aluminum alloy and CFRP. The metal layer is an aluminum alloy with copper, which has good mechanical properties (high strength and fatigue resistance) and good machinability. The material has the following mechanical properties: density of 2.78 kg/m^3^, hardness of 120 HB, ultimate tensile strength of 483 MPa, yield strength of 325 MPa, modulus of elasticity of 73.1 GPa, fatigue strength of 138 MPa, shear modulus of 28 GPa. The second material forming the sandwich structure is a polymer composite reinforced with high-strength carbon fibers (60%) (Mitsubishi Chemical Advanced Materials, Heinsberg, Germany). This material is characterized by the following mechanical properties: density of 1.60 kg/m^3^, tensile strength of 1900 MPa, bending strength of 2050 MPa, modulus of elasticity of 135 GPa, interlaminar shear strength of 85 MPa. The composite materials were formed by combining 28 layers of [0°/90°] carbon fiber twill using a Scholz autoclave (Scholz, Coesfeld, Germany). The shape and dimensions of the test samples are shown in Figure 1.

The materials making up the structure were joined together using a bonding process. A two-component epoxy adhesive (Scotch-Weld EC-9323 B/A, 3M, Saint Paul, MN, USA) was used to bond the materials at a weight ratio of 100:27. The curing process of the samples took place in a vacuum bag at a pressure of 0.1 MPa for a period of 24 h. The samples were then seasoned for 14 days under ambient conditions The polymerization and seasoning processes were carried out at 23 °C and 35% humidity. Bonded together were 2 and 3 plates of 500 × 500 × 6 mm, respectively (in the case of II-layer structure, 1 metal plate and 1 composite plate, and for III-layer structure, 2 metal plates forming the faces and 1 composite plate forming the core), which were cut into the samples shown in Figure 1 using waterjet technology. In order to remove burrs and irregularities after cutting, the samples were pre-machined by milling them to 60 × 120 × 12 mm and 60 × 120 × 18 mm dimensions.

**Figure 2:**
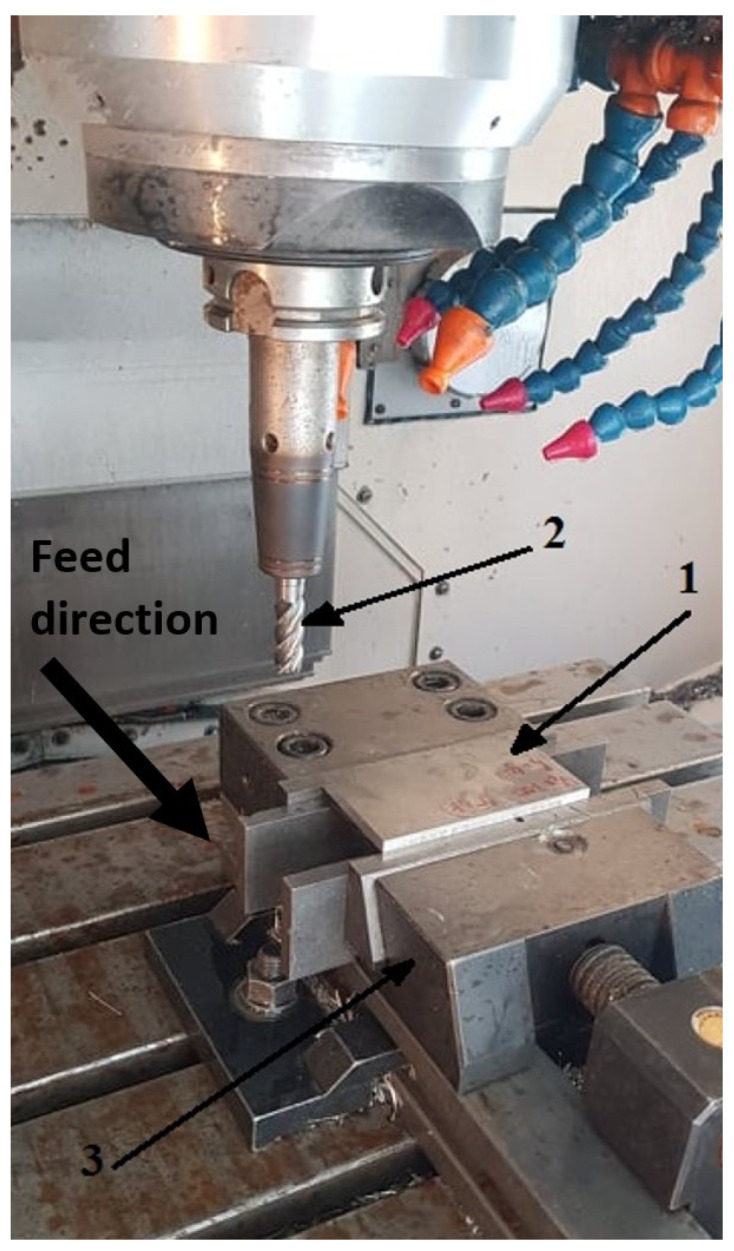
Clamping of the sample during the milling process: 1—workpiece, 2—tool, 3—machine vice.

In this study, a peripheral concurrent milling process was used with an AVIA VMC 800 HS machining center (AVIA, Warsaw, Poland). Due to the presence of a composite material, it was decided to machine without using a machining fluid. Figure 2 shows the clamping of the sample during the milling process.

The machining was carried out using the following cutting parameters: cutting speed v_c_ = 300 m/min, feed f_z_ = 0.08 mm/blade, axial depth of cut a_p_ = 12 mm for II-layer structure and a_p_ = 18 mm for III-layer structure. The values of the v_c_ and f_z_ parameters were chosen as the average value recommended by the cutting tool manufacturer and based on experience from previous studies [35,37,39]. The value of the a_p_ parameter was chosen so that the tool could simultaneously machine all the layers of the structures. The variable cutting parameter was the radial depth of cut (a_e_), which took the values of 0.5 mm, 1.0 mm, 2.0 mm, and 3.0 mm, respectively.

The type of cutting tool, due to the specific nature of the workpiece, was selected based on the possibility of machining of aluminum alloys and fiber composites. The milling process was carried out using a two-blade, shank, uncoated milling cutter of the Garant brand (Hoffman Group, Munich, Germany) with working part diameter D_c_ = 12 mm, working part length L_s_ = 20 mm, total length L = 70 mm, cutting edge helix angle λ = 45°, rake angle γ = 16°, chamfer in the corner 45° and chamfer length equal to 0.12 mm. The milling cutter was made of fine-grained K10F carbide (90% WC, 10% Co). The tool was chosen for its versatility—it can be used for both aluminum alloys and polymer composites.

**Figure 3:**
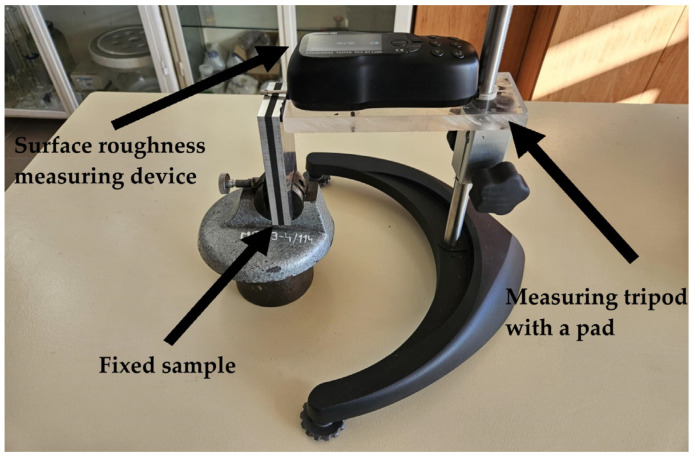
Surface roughness measurement station.

Each sample was processed three times—the analyzed results were the arithmetic average of the measurements of the three samples, and the surface roughness result for each sample was the arithmetic average of the readings at the three measurement points. The surface roughness parameters were measured using a PCE-RT 1200 surface roughness measuring device (PCE Holding AG, Meschede, Germany). The device performs the surface roughness measurements with a diamond stylus tip using the contact method [40]. All surface roughness measurements were made using the following values: evaluation length l_n_ = 0.8 mm, sampling length l_r_ = 4.0 mm, and tracing length l_t_ = 4.8 mm. The surface roughness station with the fixed sample is shown in Figure 3.

**Figure 4:**
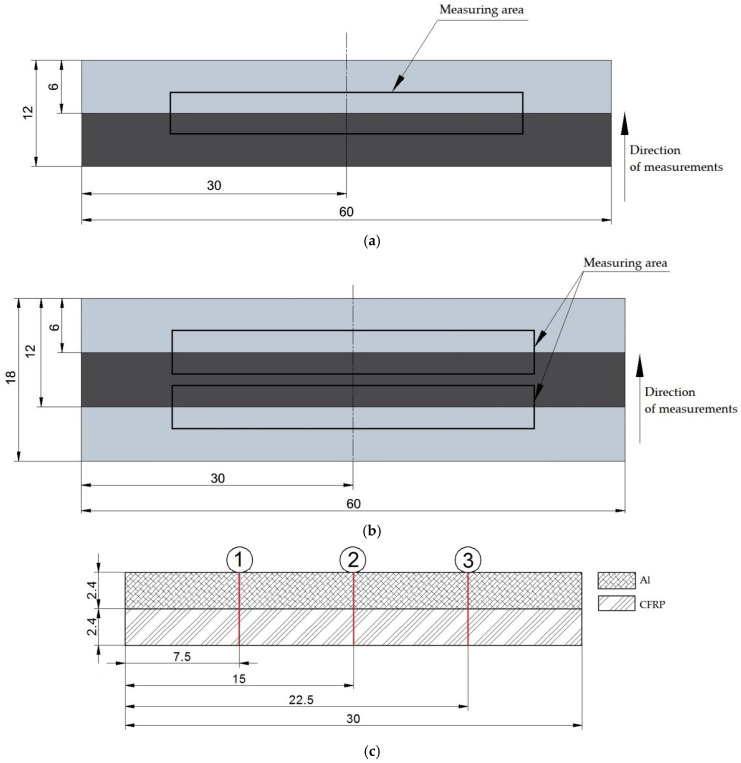
Schematic of the overall surface roughness measurement area: (**a**) location on II-layer structure; (**b**) location on III-layer structure; (**c**) measurement points considered (1, 2, 3).

**Figure 5:**
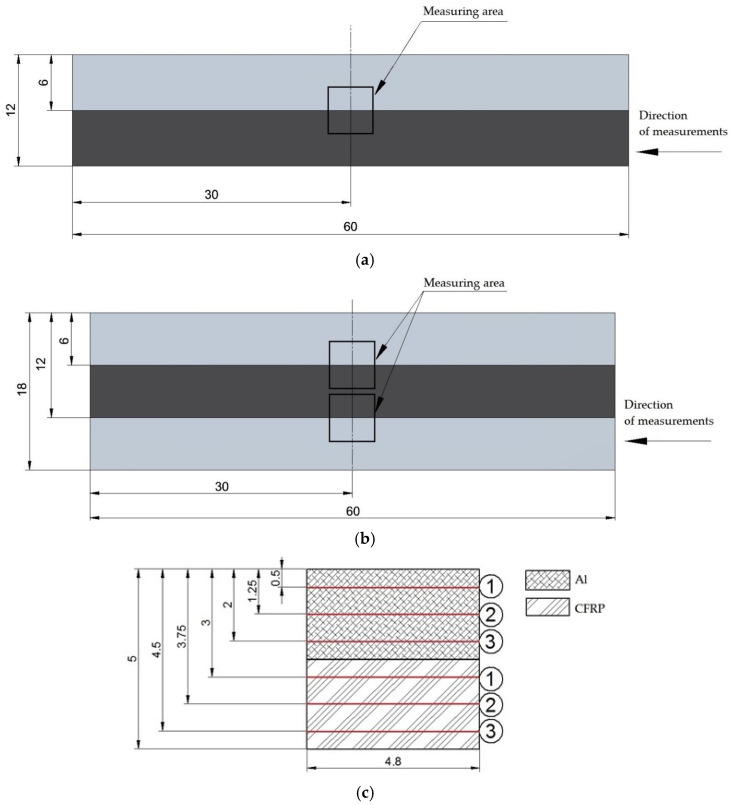
Schematic of the measurement area for individual layers: (**a**) location on II-layer structure; (**b**) location on III-layer structure; (**c**) measurement points considered (1, 2, 3).

In the experiment, the surface roughness was measured twice: the overall surface roughness of the sandwich structures (Figure 4) and the surface roughness of the individual layers that make up the samples (Figure 5). Such separation, in addition to evaluating the overall surface roughness after the milling process, made it possible to use the I_R_ index to assess the surface homogeneity of the analyzed samples. Figure 4 and Figure 5 show the directions of the measurements and the location of the measurement areas.

For measurements of the overall surface roughness of the III-layer structure, due to the limited measuring area and the dimensions of the structure (the thickness of the inner layer is 6 mm and exceeds the value of the tracing length), two measuring areas were determined (Figure 4b). The analyzed surface roughness value was the arithmetic average of the measurements from the two measurement sections. Failure to do so would result in a longer measurement distance on the CFRP surface for the III-layer structure than for the II-layer structure. This would lead to distorted results.

The surface quality was defined by the Ra (arithmetic mean deviation of the assessed profile) and Rz (maximum height of the profile) [41] surface roughness parameters and the I_R_ surface homogeneity index, which was proposed in [39]. This index is used to evaluate the surface quality of the two materials forming the layered material and takes the following form:

(1) $$I_{R}=\left|\frac{R_{Al}-R_{CFRP}}{R_{Al}+R_{CFRP}}\right|$$

where

I_R_—surface homogeneity index,

R_Al_—value of surface roughness parameter measured for aluminum alloy,

R_CFRP_—value of surface roughness parameter measured for CFRP.

This index is the difference between the surface roughness of the individual layers, which was related to the total surface roughness of the structure. Thus, this index makes it possible to assess the surface homogeneity of the sandwich structure—it informs on the achievement of similar values of surface roughness parameters for the two materials that make up the sandwich structure. The technique usually aims to make the overall surface roughness of sandwich structures as homogeneous as possible, i.e., to have similar values of surface roughness parameters for all layers. Analyzing the structure of the I_R_ index, it can be seen that the higher difference between the surface roughness parameter obtained for aluminum alloy and CFRP results in a lower homogeneous surface and a higher I_R_ value. Therefore, for the purpose of predicting the surface homogeneity of sandwich structures, it should be assumed that the lower I_R_ value, the higher surface homogeneity of the construction.

## 3. Results

**Figure 6:**
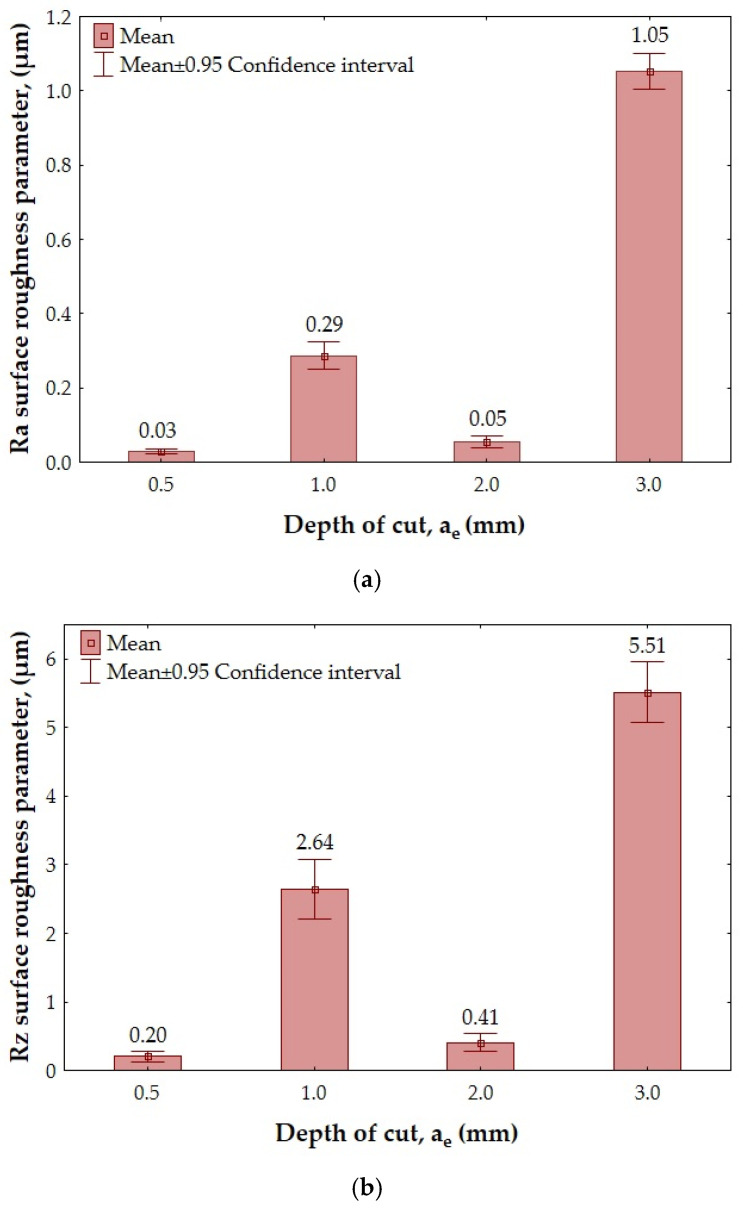
The overall surface roughness after milling of II-layer sandwich structure depending on the depth of cut a_e_: (**a**) Ra parameter; (**b**) Rz parameter.

**Figure 7:**
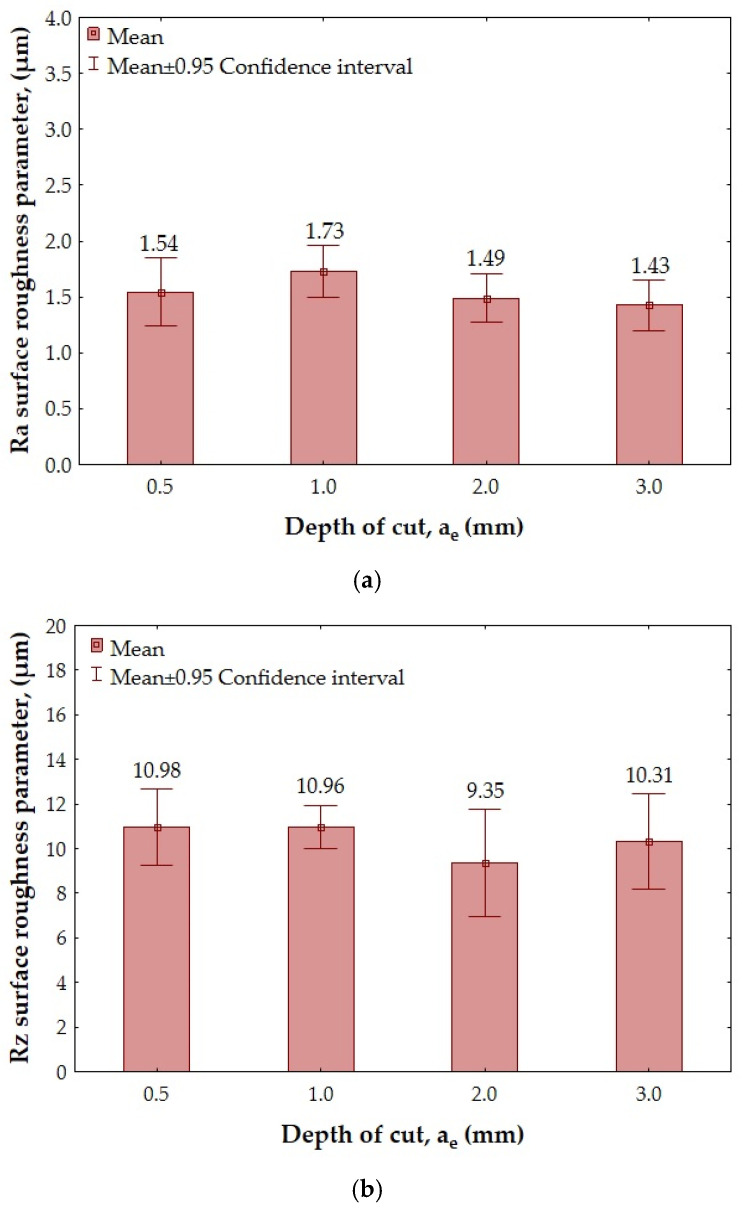
The overall surface roughness after milling of III-layer sandwich structure depending on the depth of cut a_e_: (**a**) Ra parameter; (**b**) Rz parameter.

Figure 6 and Figure 7 show the results of the overall surface roughness measurements for the II- and III-layer sandwich structures.

In the case of the Ra surface roughness parameter obtained for the II-layer structure (Figure 6a), the minimum value (Ra = 0.03 µm) was obtained after using a depth of cut a_e_ = 0.5 mm. The maximum value of this parameter (Ra = 1.05 µm) was observed after milling with a depth of cut a_e_ = 3.0 mm. Analyzing the results obtained for the Rz surface roughness parameter (Figure 6b), it can be seen that the lowest (Rz = 0.20 µm) and the highest (Rz = 5.51 µm) values of this parameter were obtained for analogous depths of cut as for the Ra surface roughness parameter.

Analyzing the values of the Ra surface roughness parameter measured for the III-layer structure (Figure 7a), it can be seen that the lowest value (Ra = 1.43 µm) was obtained for a depth of cut a_e_ = 3.0 mm. The highest value of this parameter (Ra = 1.73 µm) was observed after milling with a depth of cut a_e_ = 1.0 mm. The achieved values of the Ra surface roughness parameter in this case were similar. The lowest value of the Rz surface roughness parameter (Rz = 9.35 µm—Figure 7b) was achieved for a depth of cut a_e_ = 2.0, while the highest (Rz = 10.98 µm) was achieved for a_e_ = 0.5 mm.

**Figure 8:**
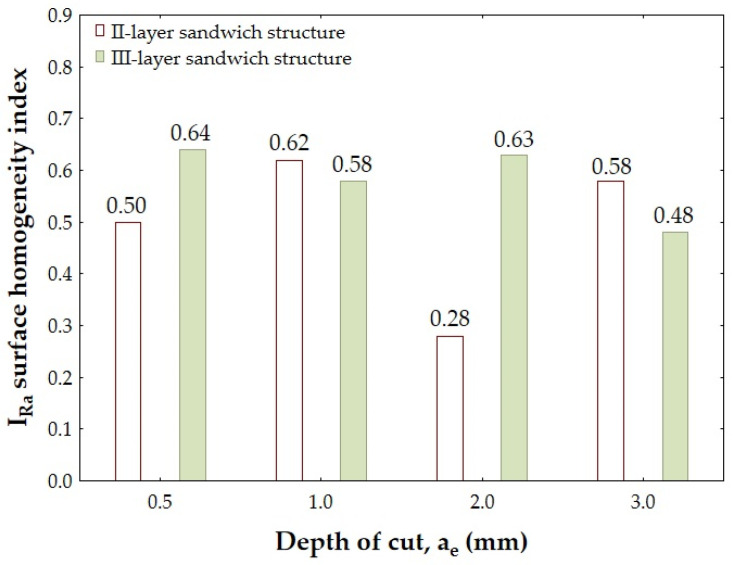
I_Ra_ surface homogeneity index.

**Figure 9:**
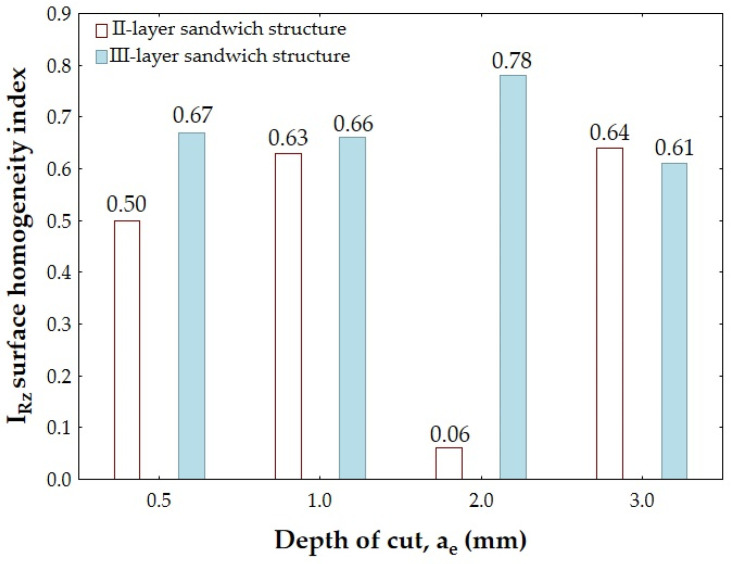
I_Rz_ surface homogeneity index.

Figure 8 and Figure 9 show the values of the I_Ra_ and I_Rz_ indices formed from the values of the Ra and Rz surface roughness parameters measured separately on the surface of the aluminum alloy and the CFRP.

The lowest value of the I_Ra_ index denoting the best surface homogeneity (I_Ra_ = 0.28) was obtained for the II-layer structure using a depth of cut a_e_ = 2.0 mm, while the highest value of this index denoting the structure with the worst surface homogeneity (I_Ra_ = 0.64) was obtained for the III-layer structure and depth of cut a_e_ = 0.5 mm. In the analyzed case, it is difficult to determine the clear impact of the a_e_ parameter on the achieved I_Ra_ values—analyzing the data, one can see alternating increases and decreases in the value of this index.

For the II-layer structure, the lowest value of the I_Ra_ index was obtained using the parameter a_e_ = 2.0 mm, while the highest value was obtained after using the depth of cut a_e_ = 1.0 mm. For the III-layer structure, the lowest value of the analyzed surface homogeneity index was observed using the parameter a_e_ = 3.0 mm and the highest with a_e_ = 0.5 mm.

Figure 9 shows the values of the I_Rz_ index formed from the values of the Rz surface roughness parameter measured separately on the surface of the aluminum alloy and the CFRP.

Analyzing the results shown in Figure 9, it can be seen that the lowest value of the surface homogeneity index formed on the basis of the Rz surface roughness parameter (I_Rz_ = 0.06) was obtained for the II-layer sandwich structure and the depth of cut a_e_ = 2.0 mm. In this case, such a low value of the I_Rz_ index confirms the achievement of a very homogeneous surface quality of the sandwich structure—very similar values of the Rz surface roughness parameter were obtained on the surface of the aluminum alloy as well as the CFRP. The highest value of the I_Rz_ index (I_Rz_ = 0.78) was observed for the III-layer structure and the depth of cut a_e_ = 2.0 mm.

Figure 8 and Figure 9 show a similar development of the I_Ra_ and I_Rz_ indices—except for the depth of cut a_e_ = 1.0 mm, where a higher I_Ra_ value was obtained for the III-layer structure. For the II-layer sandwich structure, the lowest I_Rz_ value was obtained with a_e_ = 2.0 mm, while the highest value was obtained for a_e_ = 3.0 mm. For the III-layer structure, the lowest value of the I_Rz_ index was noted after the cutting using the parameter a_e_ = 3.0 mm and the highest value of this index for a_e_ = 2.0 mm.

A multifactor analysis of variance ANOVA was performed to determine the effect of the independent variables studied on the overall surface roughness of the samples. A significance interval of α = 0.05 was adopted. The statistical analysis was performed using the Statistica 13 software (TIBCO Software, Palo Alto, CA, USA). The results of the analysis are shown in Table 1 and Table 2.

Based on the statistical analysis, it was noted that all independent variables had a statistically significant effect on the Ra surface roughness parameter. The number of the layers (II- or III-layer structures) had the highest influence (F = 374.64, *p*-value < 0.01—Table 1) on the achieved values of the Ra surface roughness parameter, followed by the A × B interaction (F = 19.75, *p*-value < 0.01), and the lowest influence, but still statistically significant, was the depth of cut (F = 12.54, *p*-value < 0.01). In this case, the *p*-value < 0.01 achieved for the A × B interaction shows that the depth of cut and the number of layers interact in influencing the values of the Ra surface roughness parameter. In such a situation, detailed simple effects should not be considered separately.

For the Rz surface roughness parameter (Table 2), the statistical analysis showed that the number of the layers had only a statistically significant effect on the achieved values of this parameter (F = 83.64, *p*-value < 0.01). The depth of cut and the A × B interaction were statistically insignificant (*p*-value > 0.05). In this case, it should be assumed that the different values of the Rz surface roughness parameter obtained for the different depths of cut and the number of the layers are 95% due to chance.

## 4. Discussion

In this paper, the effect of the depth of cut and the number of the layers on the surface roughness and the surface homogeneity after the milling process was studied. Based on the obtained results, it was noted that the a_e_ parameter affects the independent variable—in the case of the II-layer structure, the use of the higher value of the a_e_ parameter (a_e_ = 3.0 mm) resulted in the highest value of the Ra surface roughness parameter, while the use of the lowest value of the a_e_ parameter (a_e_ = 0.5 mm) resulted in a decrease in the surface roughness. It was also presented by Miah et al. [42]. An analogous result was obtained for the Rz surface roughness parameter. Similar conclusions were reached by Rodrigo et al. [43]. They showed that the use of the depth of cut a_e_ < 0.9 mm when cutting CFRP results in a lower post-machining surface roughness. This is due to the fact that a higher value of the a_e_ parameter results in increasing cutting forces, and it leads to obtaining a poorer surface quality (higher surface roughness) [44,45,46].

On the other hand, for the III-layer structure, the highest value of the Ra surface roughness parameter was obtained for a_e_ = 1.0 mm, and the lowest for a_e_ = 3.0 mm. This may be caused by using too low value of the a_e_ parameter—when machining with too small a depth of cut, the workpiece was compressed rather than cut. The use of too low value of the depth of cut (less than the value of a single layer of CFRP) also results in a deterioration of the surface quality after the cutting process as noted by El-Hofy et al. [29]. The situation was different for the Rz surface roughness parameter—the highest value of this parameter was obtained for a_e_ = 3.0 mm, and the lowest for a_e_ = 2.0 mm. The lack of the expected increase in the surface roughness with the increasing the depth of cut, which was also observed in [35], may also be due to the occurrence of typical forms of damage of the fiber composite, e.g., undercut fibers, pulled fibers, chipping of the matrix, but also the pressing of metal chips into the composite layer. A higher surface roughness was obtained for the III-layer sandwich structure than for the II-layer construction, but in most cases a lower spread of the results was observed for the III-layer structure. The higher surface roughness for the III-layer structure may be due to the additional layer of the aluminum alloy, as the surface area on which the hard, abrasive carbon fibers interact increases. Furthermore, the additional metal layer increases the volume of chips formed during machining, which are very often forced into the surface of the CFRP having a lower density. The additional layer of metal material provides more rigidity to the structure, so it is likely that in this case the effect of the a_e_ parameter on the surface quality after the milling process is not statistically significant. In addition, when machining of the III-layer structure, it was necessary to use a higher overhang tool length than when cutting the II-layer structure. This resulted in lower machining stability (stiffness of the tool) and the occurrence of higher vibrations, which contributed to the higher values of the analyzed surface roughness parameters [47].

Comparing the values of the I_Ra_ and I_Rz_ surface homogeneity indices obtained for the considered structures, it was noted that lower values were obtained for the II-layer structure in most cases, which indicates higher surface homogeneity. This is very important from the point of view of the future assembly of the structure into large-scale sandwich structures and because of the possibility of applying coatings (higher surface homogeneity means better adhesion of the coating and obtaining a more desirable visual effect). Thus, the proposed I_R_ index can be used to evaluate the surface quality of sandwich structures through the distribution of the defects after machining. A limitation of the use of this index may be the occurrence of typical forms of damage in polymer composites, such as local pulling and undercutting of the fibers. Such situations can lead to results with high dimensional inaccuracy and suggest incorrect surface homogeneity of a component. To avoid such a situation, it would be advisable to consider specifying a minimum number of measurements (or measurement points) that would produce the most reliable results. The indicator presented in the paper could also be improved by introducing into the formula a restriction based on the required manufacturing tolerance of the sandwich structure. In such a case, the I_R_ index would be an effective tool for assessing the surface homogeneity of both small-sized components and large-scale sandwich structures.

## 5. Conclusions

Based on the research, the following conclusions were made:

(1) There were different values of the Ra and Rz surface roughness parameters on the surface of the aluminum alloy and the CFRP, indicating the different machinability and the surface inhomogeneities of the sandwich structure. For this reason, the hybrid sandwich structures are classified as difficult-to-cut materials.
(2) The obtained values of the analyzed surface roughness parameters varied depending on the depth of cut and number of the layers. The statistical analysis showed that in the case of the Ra surface roughness parameter, the number of the layers influenced the surface roughness to the greatest extent, while the depth of cut had the least effect. The Rz surface roughness parameter was statistically significant, influenced only by the number of the layers of the machined structure.
(3) Higher values of the surface roughness parameters were obtained for the III-layer structure than after machining of the II-layer structure.
(4) The lowest value of the surface roughness parameters (Ra and Rz surface roughness parameters) was obtained for the II-layer sandwich structure and depth of cut a_e_ = 0.5 mm. The highest surface roughness was obtained for the III-layer structure and a_e_ = 1.0 mm (Ra surface roughness parameter) and a_e_ = 3 mm (Rz surface roughness parameter).
(5) The most homogeneous surface (the lowest values of the I_Ra_ and I_Rz_ indices) was obtained for the II-layer structure and depth of cut a_e_ = 2.0 mm. The least homogeneous surfaces were observed for the III-layer sandwich structure and the parameter a_e_ = 0.5 mm (I_Ra_ surface homogeneity index) and a_e_ = 2.0 mm (I_Rz_ surface homogeneity index).

The obtained results indicate the desirability of performing a more complete analysis on the surface quality of the hybrid sandwich structures after the machining process. One of the most important issues is the determination of acceptable post-machining surface homogeneity based on the proposed I_R_ index. The next stage of research may be to conduct experiments for other types of polymer composites, especially GFRP (Glass Fiber-Reinforced Polymer). When considering directions for further work, it would be necessary to perform a comparative analysis of the results obtained with the effects obtained after milling with a diamond tool (PCD milling cutter). The subject of further research and analysis could also be the evaluation of the influence of other geometric features of the tool on the quality of the hybrid sandwich structure after the milling process.

## Figures and Tables

**Table 1:** Multifactor ANOVA analysis of variance for Ra surface roughness parameter.

Source	SS	Df	MS	F	*p*-Value
A: depth of cut a_e_	1.15	3	0.38	12.54	<0.01
B: number of layers	11.41	1	11.41	374.64	<0.01
A × B interaction	1.80	3	0.60	19.75	<0.01
Error	0.85	28	0.03		
Total	15.21	35			

**Table 2:** Multifactor ANOVA analysis of variance for Rz surface roughness parameter.

Source	SS	Df	MS	F	*p*-Value
A: depth of cut a_e_	40.33	3	13.44	2.30	0.10
B: number of layers	489.44	1	489.44	83.64	<0.01
A × B interaction	40.40	3	13.47	2.30	0.10
Error	163.84	28	5.85		
Total	734.84	35			

## Data Availability

The raw data supporting the conclusions of this article will be made available by the authors on request.
